# Mapping anomalous dispersion of air with ultrashort mid-infrared pulses

**DOI:** 10.1038/s41598-017-01598-3

**Published:** 2017-05-18

**Authors:** A. V. Mitrofanov, A. A. Voronin, D. A. Sidorov-Biryukov, M. V. Rozhko, E. A. Stepanov, A. B. Fedotov, V. Shumakova, S. Ališauskas, A. Pugžlys, A. Baltuška, A. M. Zheltikov

**Affiliations:** 1 Russian Quantum Center, ul. Novaya 100, Skolkovo, Moscow Region 143025 Russia; 2 Physics Department, International Laser Center, M.V. Lomonosov Moscow State University, Moscow, 119992 Russia; 3 Kurchatov Institute National Research Center, Moscow, 123182 Russia; 4 Institute of Laser and Information Technologies, Russian Academy of Sciences, Shatura, Moscow Region 140700 Russia; 5 Department of Physics and Astronomy, Texas A&M University, College Station, TX, 77843 USA; 6 Kazan Quantum Center, A.N. Tupolev Kazan National Research Technical University, Kazan, 420126 Russia; 7 Photonics Institute, Vienna University of Technology, Gusshausstrasse 27-387, 1040 Vienna, Austria

## Abstract

We present experimental studies of long-distance transmission of ultrashort mid-infrared laser pulses through atmospheric air, probing air dispersion in the 3.6–4.2-μm wavelength range. Atmospheric air is still highly transparent to electromagnetic radiation in this spectral region, making it interesting for long-distance signal transmission. However, unlike most of the high-transmission regions in gas media, the group-velocity dispersion, as we show in this work, is anomalous at these wavelengths due to the nearby asymmetric-stretch rovibrational band of atmospheric carbon dioxide. The spectrograms of ultrashort mid-infrared laser pulses transmitted over a distance of 60 m in our experiments provide a map of air dispersion in this wavelength range, revealing clear signatures of anomalous dispersion, with anomalous group delays as long as 1.8 ps detected across the bandwidth covered by 80-fs laser pulses.

## Introduction

Atmospheric optics is one of the earliest fields not only in optics, but among all the natural sciences^1, 2^. As a branch of knowledge dealing with an observation and explanation of the colors of the sky, rainbows, halos, and mirages, it was exclusively concerned with the visible light and the visible spectral range, where atmospheric air is highly transparent. Systematic studies of radiation propagation in Earth’s atmosphere, motivated by the needs of observational astrophysics and performed with an ever-increasing experimental accuracy over many centuries^3^, helped achieve a detailed quantitative understanding of atmospheric transmission in the visible region.

In the era of ultrafast laser technologies, enabling the generation of high-power ultrashort field waveforms within an ultrabroad spectral range from the visible to the mid-infrared, a deeper understanding of the group-velocity dispersion (GVD) of atmospheric air is needed. This call includes a quest for anomalous-GVD ranges as a top priority for long-distance signal transmission and remote sensing of the atmosphere. Anomalously dispersive materials play a very special role in ultrafast optical science. Acting jointly with optical nonlinearity, anomalous dispersion gives rise to a vast variety of soliton phenomena^4^. In its one-dimensional version, this type of nonlinear dynamics has long been known in fiber optics^5^, enabling long-distance signal transmission and fiber-optic communications^6^, as well as short-pulse generation in fiber lasers^7^. Pulse compression in anomalously dispersive fibers^5^ has recently been extended to fiber-format subcycle pulse generation^8^. In anomalously dispersive bulk solids, spatial localization provided by spatial self-action and laser filamentation can be combined with temporal field confinement due to a solitonic dynamics of laser pulses^9^. With a careful optimization, this spatiotemporal field localization in anomalously dispersive solids enhances compression of high-peak-power mid-infrared laser pulses^10^ and can help generate light bullets as a part of laser-induced filamentation^11^. If extended to atmospheric air, these regimes would open unique opportunities for a long-distance transmission of high-peak-power laser pulses and remote sensing of the atmosphere. However, because of a complicated behavior of atmospheric refractivity within and near molecular absorption lines, identifying the ranges where atmospheric air would be both anomalously dispersive and still transparent is anything but trivial^12^.

Here, we present experiments on long-distance transmission of ultrashort mid-infrared laser pulses through atmospheric air, probing air dispersion in the 3.6–4.2-μm wavelength range. Atmospheric air is still highly transparent to electromagnetic radiation in this spectral region, making it interesting for long-distance signal transmission. However, unlike most regions of high transmission in gas media, dispersion anomalies are possible at these wavelengths due to the nearby asymmetric-stretch rovibrational band of atmospheric CO_2_. Our experiments reveal clear signatures of anomalous GVD in this wavelength range, with group delays as long as 1.8 ps detected across the bandwidth covered by 80-fs laser pulses.

To analyze the dispersion of atmospheric air in the 3.6–4.2-μm wavelength range, we calculate the refractive index of air as^13^

1 $$n(\omega )\approx 1+\frac{{e}^{2}}{2{m}_{e}{\varepsilon }_{0}}\sum _{k,i}{N}_{k}\frac{{f}_{ki}({\omega }_{ki})}{2{\omega }_{ki}}\{{[{D}_{+}(\omega )]}^{-1}-{[{D}_{-}(\omega )]}^{-1}\}+{n}_{vis}(\omega )$$

where *ω* is the frequency, *N*_k_ is the density of molecules of sort *k*, *ω*_*ki*_, *Γ*_*ki*_, *f*_*ki*_ are the frequency, the linewidth, and the oscillator strength of the *i*th resonance in the spectrum of molecules of sort *k*, *m*_e_ and *e* are the electron mass and charge, *ε*_0_ is the dielectric permittivity of vacuum, *D*_+_(*ω*) = *ω* + *ω*_*ki*_ − *iΓ*_*ki*_/2, *D*_−_(*ω*) = *ω* − *ω*_*ki*_ − *iΓ*_*ki*_/2, and *n*_vis_(*ω*) is the refractive index of air in the visible–near-infrared range, calculated with the standard formula^14–16^ *n*_vis_(*λ*) = *B*_1_(*C*_1_ − *λ* ^−2^)^−1^ + *B*_2_(*C*_2_ − *λ* ^−2^)^−1^, with *B*_1_ = 0.05792105 µm^−2^, *B*_2_ = 0.00167917 µm^−2^, *C*_1_ = 238.0185 µm^−2^, and *C*_2_ = 57.362 µm^−2^ ^17^.

In the full model of air refractivity, we calculate the refractive index of air with Eq. (1) including the entire HITRAN-database manifold of molecular transitions in air^18^. The GVD of atmospheric air can then be found as *k*_2_ = ∂^2^ *k*(*ω*)/∂*ω* ^2^, where *k*(*ω*) = *ωn*(*ω*)/*c*, *c* is the speed of light in vacuum, and *n*(*ω*) is calculated using Eq. (1). Dispersion of atmospheric air within the 3.5–4.2-μm wavelength range is dominated by the 00°01–00°11 asymmetric-stretch rovibrational band of atmospheric CO_2_ (here, the *v*_1_ *v*_2_^*l*^ *v*_3_ *r* notation is adopted for a superposition of *v*_1_ symmetric-stretch, *v*_2_ bending, and *v*_3_ asymmetric-stretch vibrations with an angular momentum *l*, and Fermi-resonance perturbation parameter *r*). The frequencies of rovibrational transitions within the P and R branches of this molecular band are given, within the standard approximation^15^, by *ω*_R,P_(*J*) = *ω*_1_(*J* ± 1) − *ω*_0_(*J*) with *ω*_0,1_(*J*) ≈ *G*_0,1_ + *B*_0,1_(*J* + 1), where *G*_*v*_ are the vibrational terms and *B*_*v*_ are the rotational constants of a molecule, *J* and *v* being the rotational and vibrational quantum numbers, respectively. For the 00°01–00°11 mode of CO_2_, *G*_0_ = 0, *G*_1_ ≈ 442.80300 rad/ps, *B*_0_ ≈ 0.073554528 rad/ps, and *B*_1_ ≈ 0.072974425 rad/ps.

The intensities of individual spectral lines within the R and P branches of the 00°01–00°11 CO_2_ band, related to the oscillator strength by *f*_*ki*_ = *f*_R,P_(*J*, *T*) = (4*m*_e_ *c* ^2^ *ε*_0_/*e* ^2^)*S*_R,P_(*J*, *T*), are given by ref. 19

2 $${S}_{R,P}(J,T)=\frac{{\omega }_{R,P}}{{\tilde{\omega }}_{0}}{S}_{0}(T){L}_{R,P}(J)[\frac{\exp (-\hslash {\omega }_{0}(J)/kT)}{Q(T)}]\,[1-\exp (-\frac{\hslash {\omega }_{R,P}}{kT})],$$

where *T* is the temperature, ${\tilde{\omega }}_{0}={G}_{1}-{G}_{0},{S}_{0}(T)$ is the line strength, *L*_R,P_(*J*) is the Honl–London factor, *Q*(*T*) = Σ_*J*_(2 *J* + 1)exp{−*ħ* ${\tilde{\omega }}_{0}$(*J*)/*kT*} is the rotational partition sum, *k* is the Boltzmann constant, and *ħ* is the Planck constant.

**Figure 1:**
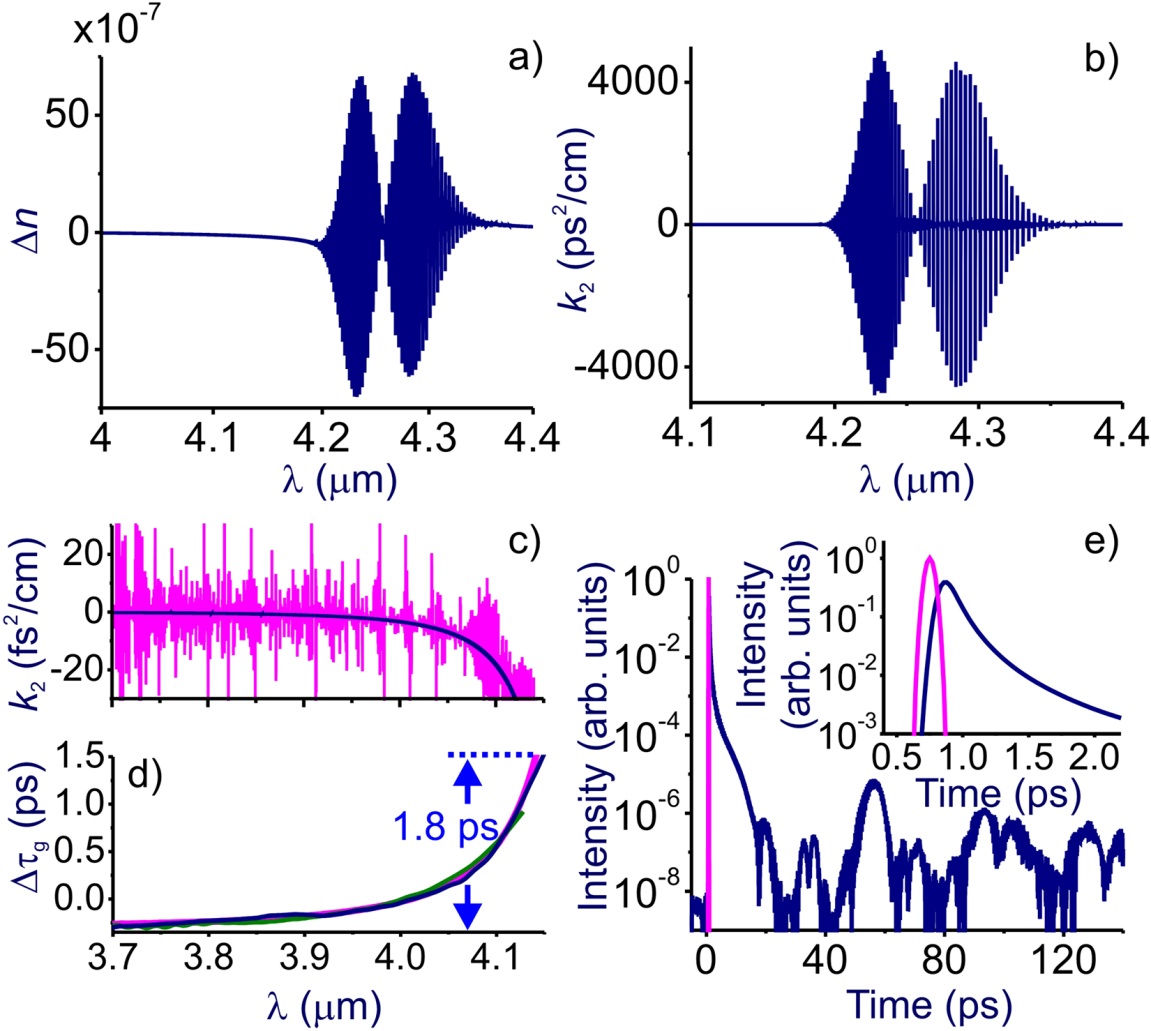
(**a**,**b**) The refractive index of air (**a**) and the group velocity dispersion *k*_2_ (**b**) calculated as a function of the wavelength using the full model of air refractivity [Eq. (1)] including the entire manifold of HITRAN-database infrared transitions in atmospheric air under normal conditions at *T* = 20 °С; Δ*n* = *n* − *n*_0_, *n*_0_ = 1.000270232 is the refractive index of air at *λ* ≈ 3.9 µm. (**c**) The blowup of the GVD profiles in the high-frequency wing of the asymmetric-stretch band of CO_2_ molecules calculated by using Eq. (1) that includes only the 00°01–00°11 band of atmospheric CO_2_ (blue line) and the full model of air dispersion with the entire manifold of HITRAN-database infrared transitions (red line). (**d**) The differential group delay Δ*τ*_g_ induced by 60 m of atmospheric air as retrieved from the experiments performed with a transform-limited (blue curve) and negatively chirped (green line) mid-infrared probe versus calculations performed with the use of the full model of air dispersion (red line). (**e**) Temporal envelope of an 80-fs, 3.9-μm laser probe transmitted through 60 m of atmospheric air calculated using Eqs (3) and (4) with the full model of air refractivity [Eq. (1)] that includes the entire manifold of molecular transitions in air from the HITRAN database (blue line). The input laser probe is shown by the red line.

In Fig. 1a and b, we plot the refractive index and the GVD of atmospheric air within the 4.2–4.3-μm 00°01–00°11 band of atmospheric CO_2_ and in the high-frequency wing of this band. Within the absorption band, that is, for wavelengths *λ* longer than 4155 nm, individual rovibrational transitions of CO_2_ give rise to rapid oscillations in the refractive index (Fig. 1a) and the GVD (Fig. 1b) of air.

It is, however, the high-frequency wing of this molecular band, stretching from approximately 3.5 to 4.2 μm, that we identify here as the region that offers unique options for the ultrafast optics of the atmosphere. Here, unlike the central part of the CO_2_ absorption band, where the attenuation length is as short as *l*_a_ ≈ 1 m at *λ* = 4.26 μm, atmospheric air is already highly transparent, with the attenuation length at our laser wavelength of 3.9 μm estimated as *l*_a_ ≈ 40 km. Remarkably, in contrast to most of the high-transmission regions in gas media, the GVD is anomalous in this region (Fig. 1b,c). This unique combination of high transmission and anomalous GVD is achieved due to the high probabilities of rovibrational transitions within this molecular band, expressed by the high integral oscillator strength of the entire band.

Due to the large *f*_R,P_(*J*, *T*) within the P and R branches of the 00°01–00°11 band of CO_2_, the anomalous GVD provided by the second term in Eq. (1) for *ω* > *ω*_R,P_(*J*) prevails over the normal GVD due to the *n*_vis_(*ω*) term, which dominates the GVD within the entire visible and most of the near-infrared range. As can be seen from Fig. 1b and c, right outside the CO_2_ absorption band, i.e., for *λ* < 4.2 μm, the net GVD remains anomalous within the bandwidth that is broad enough to support pulses as short as ~200–250 fs.

The net GVD calculated by using Eq. (1) that includes only the 00°01–00°11 band of atmospheric CO_2_ with density ${N}_{{{\rm{CO}}}_{2}}\approx 1.32\cdot {10}^{16}{{\rm{cm}}}^{-3}$ is shown by the blue line in Fig. 1c. It is seen to provide a continuously anomalous GVD everywhere within the 3.5–4.2-μm range. However, when other major molecular atmospheric constituents, primarily H_2_O, O_3_, and CH_4_, are added to the sum in *k* in Eq. (1), with their densities *N*_*k*_ taken at normal conditions (${N}_{{{\rm{H}}}_{{\rm{2}}}{\rm{O}}}$ ≈ 5.4 ∙ 10^16^ cm^−3^, ${N}_{{{\rm{O}}}_{{\rm{3}}}}$ ≈ 2.5 ∙ 10^12^ cm^−3^, and ${N}_{{{\rm{CH}}}_{{\rm{4}}}}$ ≈ 4.0 ∙ 10^13^ cm^−3^), the GVD behavior within the 3.5–4.2-μm range becomes much more complicated. Now, the high-frequency negative-GVD tail provided by the 00°01–00°11 CO_2_ band is observed against oscillatory GVD features related primarily to H_2_O, O_3_, and CH_4_ molecules (red line in the upper panel of Fig. 1c). These features are almost invisible in Δ*n*(*ω*) and *k*_2_(*ω*) in Fig. 1a and b, but show up in the blowup of the GVD profile in Fig. 1c.

To understand the significance of these small-scale oscillatory GVD features for the dynamics of ultrashort mid-infrared pulses, we examine the temporal evolution

3 $$A(\eta )={\int }_{-\infty }^{\infty }{A}_{0}(\xi )G(\eta -\xi )d\xi $$

of a Gaussian laser pulse *A*_0_(*η*) whose initial pulse width, *τ*_0_ = 80 fs, and central wavelength, *λ*_0_ = 3.9 μm, are chosen in such a way as to model laser probe pulses in our experiments. The kernel in the time-evolution integral,

4 $$G(\eta )={\int }_{0}^{\infty }\exp (i\omega t-ik(\omega )z)d\omega ,$$

includes all the absorption and dispersion features of atmospheric air as shown in Fig. 1c.

Results of these calculations clearly show (Fig. 1e) that the small-scale oscillatory GVD features in the 3.5–4.2-μm range have no effect on the dynamics of ultrashort probe pulses in our experimental conditions, as they show up on a very long, ~10-ps time scale as extremely weak features, whose intensity is about eight orders of magnitude lower than the intensity of the laser probe (Fig. 1e). As is also seen in Fig. 1e, the temporal envelope of the transmitted laser pulse on the ~10–100-ps time scale is dominated by rotational echo revivals (e.g., those centered at *η* ≈ 55 and 100 ps in Fig. 1e) – a standard behavior of a laser probe in time-resolved studies of molecular rovibrational modes^20, 21^.

**Figure 2:**
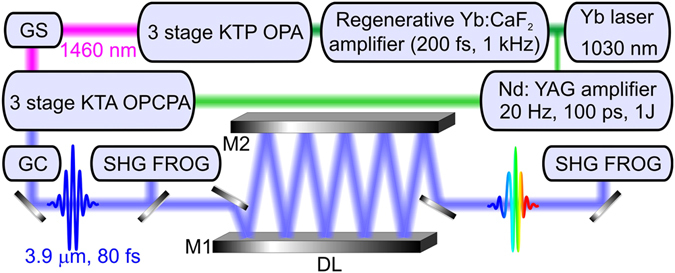
Experimental setup: GS, grism stretcher; GC, grating compressor; DL, 60-m propagation-path delay line; M1, M2, mirrors.

Ultrashort mid-IR pulses are delivered in our experiments by a laser system (Fig. 2) consisting^22, 23^ of a solid-state ytterbium laser with an amplifier, a three-stage optical parametric amplifier (OPA), a grating–prism (grism) stretcher, a Nd: YAG pump laser, a three-stage OPCPA system, and a grating compressor for mid-IR pulses. The 1-kHz, 200-fs, 1–2-mJ, 1030-nm regeneratively amplified output of the Yb: CaF_2_ laser system is used as a pump for the three-stage OPA, which generates 1460-nm pulses at its output. These 1460-nm pulses are then stretched with a grism stretcher and used as a seed in a three-stage OPCPA, consisting of three KTA crystals (Fig. 2), pumped by 100-ps Nd: YAG-laser pulses with energies 50, 250, and 700 mJ, respectively. The stretched-pulse idler-wave output of the OPCPA system has a central wavelength of 3.9 μm and an energy up to 50 mJ. Compression of these pulses using a grating compressor yields mid-IR pulses with a pulse width of 80 fs and an energy up to 30 mJ.

To provide a long propagation path of a mid-infrared beam through atmospheric air, we use an optical delay line consisting of two rectangular silver mirrors (Fig. 2). The mid-infrared beam makes six 10-m round trips, bouncing off the rectangular mirrors, thus propagating a path of *L* ≈ 60 m in atmospheric air. The input beam radius in our experiments is *r*_0_ ≈ 0.7 cm, corresponding to a Rayleigh range *l*_R_ ≈ 80 m. Spectral measurements in the mid-infrared range are performed with a homebuilt spectrometer consisting of a scanning monochromator and a thermoelectrically cooled HgCdTe detector (Fig. 2). For the spectral measurements in the ultraviolet, visible, and near-infrared ranges, OceanOptics HR4000 and NIRQuest spectrometers were employed.

Temporal envelopes and phases of mid-infrared pulses at the output of the OPCPA and behind the 60-m delay line are characterized using frequency-resolved optical gating (FROG) based on second-harmonic generation (SHG) in a 0.5-mm-thick AgGaS_2_ crystal (Fig. 2). FROG traces are measured using two identical beam replicas produced with a thin-film beam splitter, which travel equal propagation paths before reaching the NIRQuest spectrometer accurately calibrated up to a wavelength of 2130 nm.

**Figure 3:**
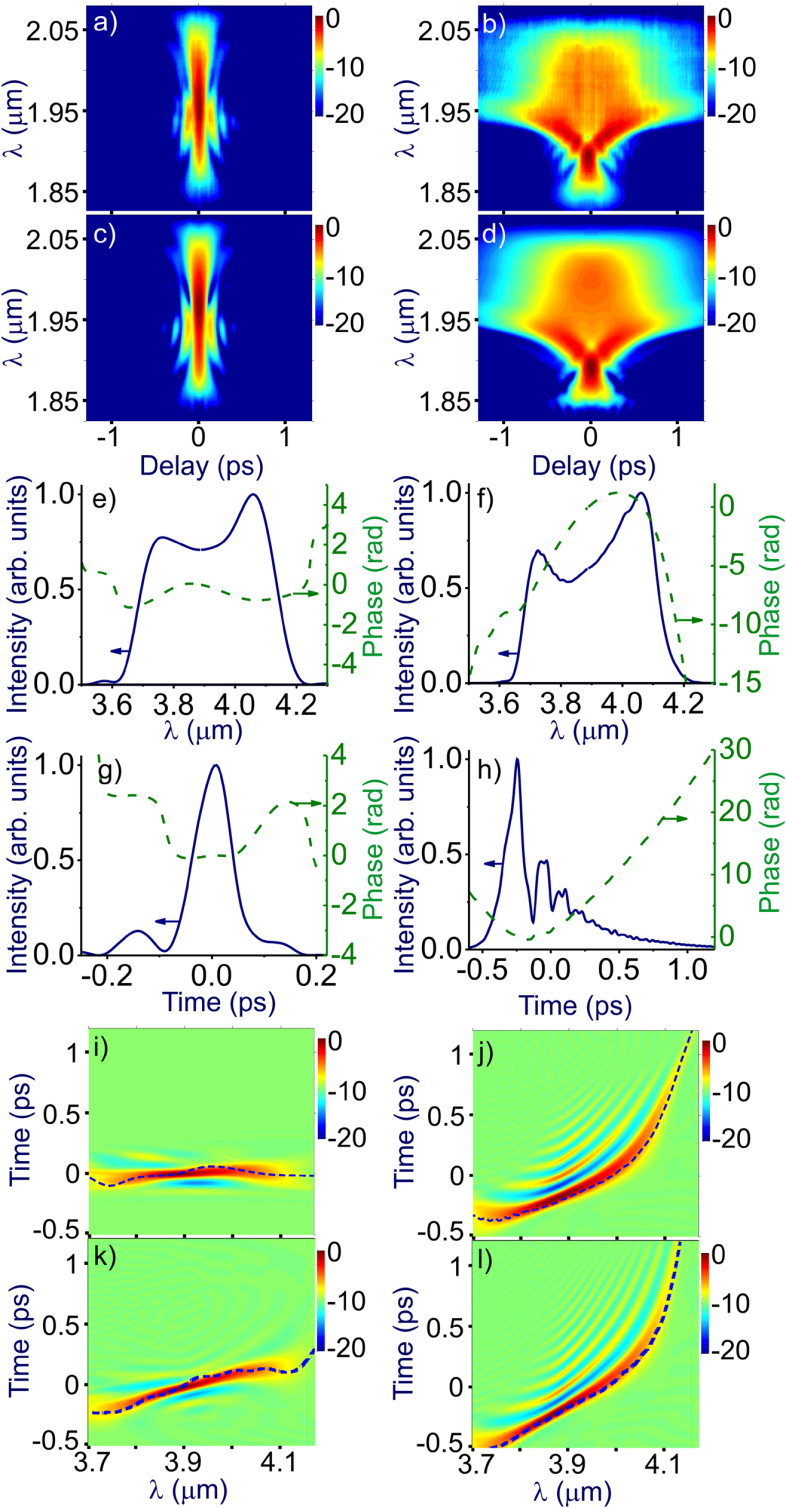
(**a–d**) Measured (**a,b**) and reconstructed (**c,d**) FROG traces of the OPCPA output (**a,c**) and the laser probe transmitted through 60 m in atmospheric air (**b,d**), (**e,f**) spectra (solid lines) and spectral phases (dashed lines), (**g,h**) temporal envelopes (solid lines) and phases (dashed lines) retrieved from the FROG traces of the OPCPA output (**e,g**) and the laser probe transmitted through 60 m in atmospheric air (**f,h**), (**i,j**) Wigner spectrograms of the OPCPA output (**i**) and the mid-IR laser probe transmitted through 60 m in atmospheric air (**j**). (**k,l**) Wigner spectrograms of the mid-IR probe with an initial chirp of −0.007 ps^2^ at the OPCPA output (**k**) and behind the 60-m delay line (**l**). The group delay is shown by the dashed line in the Wigner maps.

In Fig. 3a, we present a typical experimental FROG trace of a mid-infrared OPCPA output used in our experiments. The spectrum and the temporal envelope of these pulses are shown in Fig. 3e and g, respectively. To probe the dispersion of atmospheric air in the spectral region where anomalous GVD is expected based on our analysis, the OPCPA source was adjusted in such a way as to provide an idler-beam mid-infrared output with a spectrum stretching from 3.6 to 4.2 μm (Fig. 3e). When operated in this mode, the OPCPA delivers a mid-infrared output with a minimum transform-limited pulse width of about 80 fs (Fig. 3g). The probe pulse energy was kept below 4 mJ in our experiments to avoid phase distortions due to nonlinear phase shifts over large propagation paths in atmospheric air.

Transmission over 60 m in atmospheric air gives rise to a dispersion-induced phase shift, readily noticeable in FROG traces (Fig. 3b,d), the spectral phase profiles *φ*_t_(*λ*) retrieved from these traces (Fig. 3f,h), and especially in the Wigner spectrograms in Fig. 3j and l. To identify the sign of air GVD within the studied spectral range, measurements with a negatively chirped broadband mid-infrared probe were performed. When transmitted through atmospheric air, such a laser probe is seen to increase its chirp *φ*_t_ (cf. Fig. 3k and l). The phase shift across the spectrum of the transmitted pulse (Fig. 3l) is seen to be substantially larger than the change in the phase *φ*_0_(*ω*) related to the initial chirp of this pulse (Fig. 3k). Correspondingly, the group delay *τ*_g_ = ∂*φ*_t_/∂*ω* accumulated within the spectrum of the transmitted probe is much larger compared to the group delay *τ*_0_ = ∂*φ*_0_/∂*ω* related to the initial chirp (cf. Fig. 3k and l).

In Fig. 1d, we present the differential group delay Δ*τ*_g_ = *τ*_g_ − *τ*_0_, which isolates the group delay acquired by the probe due to atmospheric dispersion from the group delay due to the initial chirp. This allows the measurements performed with probe pulses with different initial chirps to be directly compared with each other. The plot in Fig. 1d presents three almost indistinguishable Δ*τ*_g_(*λ*) dependences. Two of these curves represent measurements performed with an almost transform limited (blue line) and negatively chirped (green line) probe pulses. The red line shows the results of calculations performed with the full model of air dispersion including the entire manifold of HITRAN-database infrared transitions. Both the overall behavior of the group delay as a function of the wavelength and the absolute values of Δ*τ*_g_ achieved in experiments (blue and green lines in Fig. 1d) agree remarkably well with theoretical predictions (red line in Fig. 1d), verifying the predictive power of the model of atmospheric dispersion used in this work. The differential group delay Δ*τ*_g_ is seen to be a monotonically growing function of the wavelength within the entire wavelength range covered by the spectrum of the laser probe, clearly indicating anomalous GVD within this wavelength range.

The absolute values of Δ*τ*_g_ achieved in experiments were found to be sensitive to the concentration of CO_2_ in the room. This observation is consistent with the assignment of negative GVD observed in our experiments to the rovibrational band of atmospheric CO_2_. To make direct comparison of calculations and experiments possible, the concentration of CO_2_ in air was measured during the experiment using a standard 7798 CO_2_ datalogger. With ${N}_{{{\rm{CO}}}_{2}}$ ≈ 1.32 ∙ 10^16^ cm^−3^ taken from the readings of the CO_2_ monitor, calculations are seen to provide a highly accurate fit for the results of Δ*τ*_g_ measurements. For this CO_2_ density, anomalous group delays as long as Δ*τ*_g_ ≈ 1.8 ps are achieved within the wavelength range from 3.70 to 4.15 μm in our experiments.

**Figure 4:**
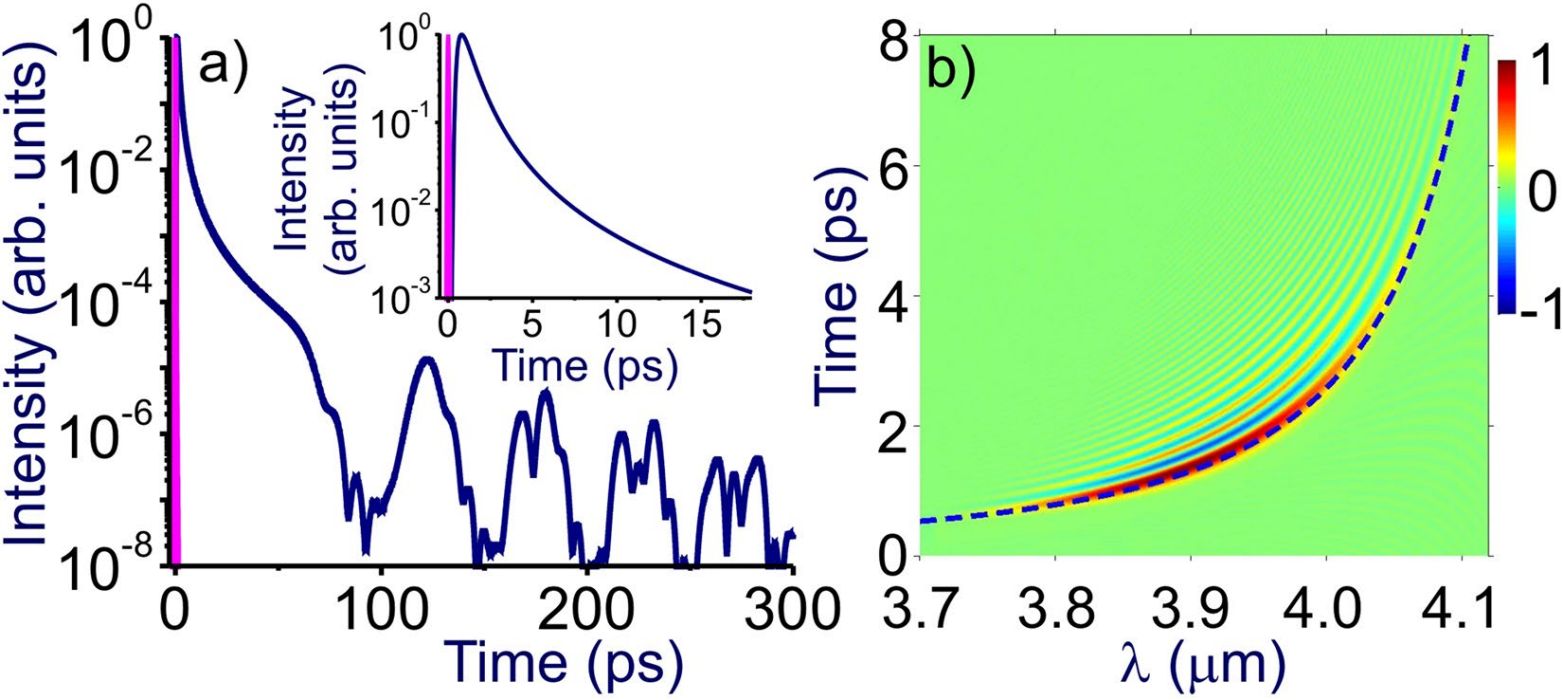
Temporal envelope (**a**) and the Wigner spectrogram (**b**) of an 80-fs, 3.9-μm transform-limited laser probe with *r*_0_ ≈ 0.7 cm transmitted through 500 m of atmospheric air calculated with Eqs (3) and (4). The input laser probe is shown by the red line. The group delay Δ*τ*_g_ is shown by the dashed line in the Wigner map.

Within longer propagation ranges, *L* > 60 m, the laser pulse continues to acquire a group delay due to the anomalous GVD of air. Although the spectrum of our laser probe is close to the CO_2_ absorption band, it still lies in the region of high atmospheric transmission. At the central wavelength of our laser probe, *λ* = 3.9 μm, the attenuation length is as long as *l*_a_ ≈ 100 km. The absorption increases in the long-wavelength tail of the laser pulse, with *l*_a_ ≈ 10 km at the wavelength *λ* = 4.17 μm, where the spectral intensity of the laser field is 20 times lower than the maximum intensity at *λ* = 3.9 μm. As a consequence, even for a propagation range as long as *L* = 500 m, only 4% of an 80-fs, 3.9-μm transform-limited pulse is lost due to atmospheric absorption. In Fig. 4a and b, we show the temporal envelope and the Wigner map of such a pulse calculated for *L* = 500 m using Eqs (3) and (4). The mid-IR probe is seen to acquire a strong chirp within such a long propagation path, which stretches the main peak in the leading edge of this pulse to several picoseconds (the inset in Fig. 4a). The signatures of rotational echo revivals are still observed in the field waveform as weak features at *η* ≈ 120, 175, 225 and 270 ps (Fig. 4a). In the long-wavelength wing of the laser probe spectrum, the chirp of the pulse is highly nonlinear (Fig. 4b) due to high-order dispersion, which rapidly increases toward the edge of the CO_2_ absorption band. As long as the peak power *P* of the laser probe (*P* < 50 GW for laser pulses used in our experiments) is lower than the critical power of self focusing, *P*_cr_ ≈ 75 GW at *λ* ≈ 3.9 μm, spatial self-action effects do not play any noticeable role in long-range beam dynamics.

The GVD of air is sensitive to the partial density of atmosphere constituent gases. This sensitivity dramatically increases near the absorption bands of the respective gas. A small change in the CO_2_ density by Δ${N}_{{{\rm{CO}}}_{2}}$ gives rise to a wavelength-dependent increment in anomalous GVD, which induces a group delay $\delta \tau ={c}^{-1}{\int }_{0}^{L}[{n}_{1}(z)-{n}_{2}(z)]dz$ between two spectral components *λ*_1_ and *λ*_2_ of a laser probe transmitted over a propagation path *L*, *n*_1_(*z*) and *n*_2_(*z*) being the group indices at *λ*_1_ and *λ*_2_, which depend on the coordinate *z* along the propagation path through the local CO_2_ density ${N}_{{{\rm{CO}}}_{2}}$(*z*). Specifically, for the 80-fs mid-IR laser probe used in our experiments, *λ*_1_ and *λ*_2_ can be defined as *λ*_1_ = 3685 nm and *λ*_2_ = 4140 nm. Then, taking an average CO_2_ density ${\bar{N}}_{C{O}_{2}}$ ≈ 1.32 ∙ 10^16^ cm^−3^, as in our experiments, and setting *L* equal to the minimum of the absorption length *l*_a_ within the range of wavelengths from *λ*_1_ to *λ*_2_, that is, *L* = *l*_a_ ≈ 30 km at *λ*_2_ = 4140 nm for mid-IR pulses in our studies, we find that a group delay *δτ* ≈ 80 fs, which can be reliably detected in the Wigner maps of 80-fs probe pulses, corresponds to an average (over *L*) variation in the CO_2_ density as small as $\delta N={L}^{-1}{\int }_{0}^{L}{N}_{C{O}_{2}}(z)dz-{\bar{N}}_{C{O}_{2}}$ ≈ 10^11^ cm^−3^, which is equivalent to a sensitivity of 4.7 ppb under normal atmospheric conditions. With the same assumptions, the accuracy of CO_2_ density measurements that can be achieved with the propagation path *L* ≈ 60 m, as in our experiments, is estimated as 2.4 ppm.

To summarize, atmospheric air has been shown to display broadband anomalous GVD in the 3.6–4.2-μm range, in the high-frequency wing of the asymmetric-stretch CO_2_ band, that is, in the region where atmospheric air is highly transparent to electromagnetic radiation. Anomalous air GVD demonstrated in this work paves the ways for long-distance signal transmission, new remote sensing techniques, and soliton pulse transformations at high levels of peak powers within long propagation paths in atmospheric air.
